# Stimulus Intensity in Left Ventricular Leads and Response to Cardiac Resynchronization Therapy

**DOI:** 10.1161/JAHA.112.000950

**Published:** 2012-10-25

**Authors:** Venkata V. Bavikati, Jonathan J. Langberg, B. Robinson Williams, Danesh Kella, Michael S. Lloyd

**Affiliations:** Emory University School of Medicine, Atlanta, GA

**Keywords:** cardiac resynchronization, congestive heart failure, pacing

## Abstract

**Background:**

Increased left ventricular (LV) stimulus intensity has been shown to improve conduction velocity and cardiac output. However, high-output pacing would shorten device battery life. Our prospective trial analyzed the clinical effects of high- versus low-output LV pacing.

**Methods and Results:**

Thirty-nine patients undergoing initial cardiac resynchronization therapy device implantation with bipolar LV leads were assigned to 3 months of either high-output LV pacing (Hi) or low-output LV pacing (Lo) in a randomized, blinded crossover fashion. Hi and Lo settings were determined with a rigorous intraoperative protocol specific to each patient. Clinical and echocardiographic data were obtained at randomization, at 3 months, and a subsequent 3 months after crossover. Mean age was 66.4±9.8 years, and mean QRS duration was 159.3±23.1 ms. Compared to baseline, both arms had significant improvements in Minnesota Living With Heart Failure score (given as mean [95% confidence interval]) (baseline versus Lo: 43.3 [35.5 to 51.1] versus 21.3 [14.6 to 28.0], *P*<0.01; baseline versus Hi: 43.3 [35.5 to 51.1] versus 23.6 [16.1 to 31.1], *P*<0.01) and 6-minute walk distance (baseline versus Lo: 692 ft [581 to 804] versus 995 ft [876 to 1114], *P*<0.01; baseline versus Hi: 699 ft [585 to 813] versus 982 ft [857 to 1106], *P*<0.01). Although both Hi and Lo arms had some echocardiographic parameters that significantly improved compared to baseline (baseline end-diastolic diameter 5.7 cm [5.5 to 6.0] versus Lo 5.5 cm [5.1 to 5.8], *P*<0.01; baseline end-systolic diameter 4.9 cm [4.6 to 5.3] versus Hi 4.7 cm [4.3 to 5.0], *P*<0.05), there were no significant differences observed when comparing the Hi- versus Lo-output arms.

**Conclusions:**

Low-output LV pacing with a relatively narrow safety margin above capture threshold affords significant improvement from baseline and is clinically equivalent to high-output LV pacing. These data support a strategy of minimizing the programmed LV safety margin to increase battery life in cardiac resynchronization therapy devices.

**Clinical Trial Registration Information:**

URL: http://www.clinicaltrials.gov. Unique identifier: NCT01060449

## Introduction

Despite the proven benefits of cardiac resynchronization therapy (CRT) with regard to risk of death and quality of life, approximately one third of appropriate CRT candidates fail to respond.^1,2,3,4^ One of several potential reasons for CRT nonresponse is suboptimal left ventricular (LV) pacing.^5,6^ Suboptimal LV pacing can include LV pacing in a discordant location that is not on the opposing side of conduction block or that is not particularly delayed and thus fails to correct the underlying dyssynchrony.^7,8,9^ Suboptimal LV pacing also can result from transient failure of the stimulus to capture myocardium. Because epicardial pacing tends to have higher thresholds than endocardial pacing, and because those with higher thresholds have been shown to have greater variability in capture threshold over time,^10^ a relatively narrow margin between capture threshold and stimulus strength can result in occasional LV noncapture and inconsistent resynchronization.

One simple yet unexplored way to partially overcome these obstacles is to increase LV pacing stimulus intensity.^11^ Increased stimulus intensity expands the virtual electrode of directly excited myocardium beyond local regions of propagation block. Because it results in a larger region of LV myocardium being directly stimulated, it potentially would increase the chance of the most dyssynchronous LV regions being stimulated.^12,13^ Additionally, transient failure to capture the LV would be less likely with increased stimulus intensity. We and others have shown faster conduction times and evidence for expanded virtual electrodes in human ventricles when LV stimulus intensity is increased.^14,15,16^

Nevertheless, the potential benefits of increased LV stimulus intensity suggested by experimental data could be outweighed by the cost of reducing device battery life. Of all approved implantable cardiac devices, the average battery longevity in CRT devices is lowest, and predictions on battery longevity historically have been overestimated.^17^ More frequent generator exchanges increase risks for patients.^18^ Therefore, the present study was designed to compare clinical measures of high (Hi) versus low (Lo) LV stimulus intensity in a double-blinded, prospective crossover in patients undergoing CRT implantation.

## Methods

**Figure 1.**
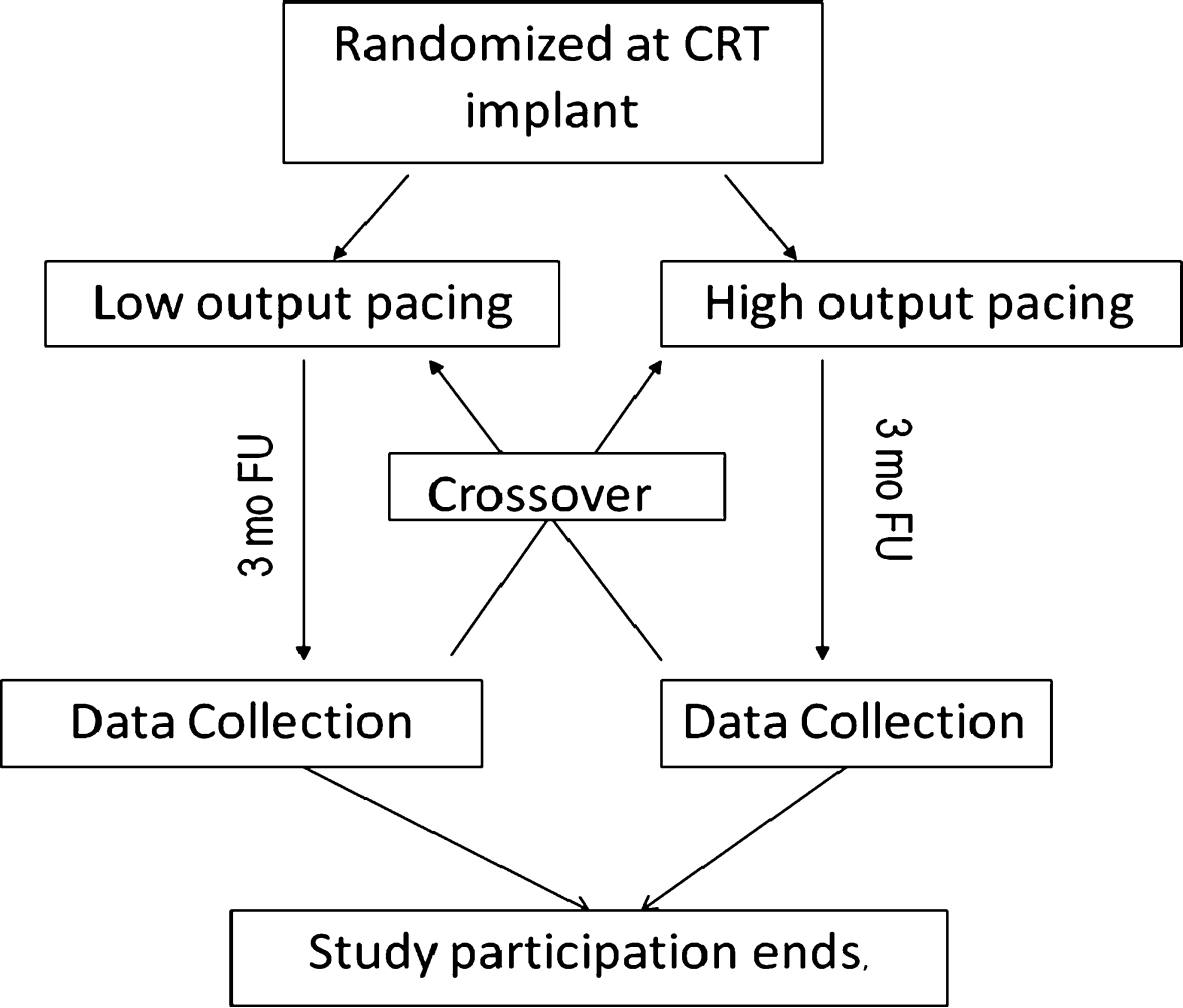
Protocol design of the SILVeR-CRT trial. FU indicates follow-up.

The Stimulus Intensity in Left Ventricular Leads and Response to Cardiac Resynchronization Therapy (SILVeR-CRT) was a single-center, randomized, double-blind, crossover design trial conducted at Emory University Hospital and approved by the Emory University institutional review board. Informed consent was obtained from all patients before enrollment. A single LV lead model (Medtronic 4196, Attain Ability) was used in all study patients. The distance between the distal (tip) and proximal (ring) electrode of this lead is 21 mm, and the surface areas of both electrodes are identical (5.8 mm^2^). The randomized crossover design of the trial was chosen to account for the effects of time on the measured variables, as well as intrapatient differences, given the modest size of the study population (Figure 1). LV lead implantation technique and location were left to the discretion of the implanting physicians.

### Hi- Versus Lo-Output Setting Determinations

The Hi and Lo stimulus intensities were contingent on LV thresholds in each study participant. Capture thresholds were obtained in a unipolar mode, first with the LV lead tip used as the cathode and ground as the anode and then with the LV lead ring used as the anode and ground as the cathode. The Lo setting was defined as 1 V above the unipolar capture threshold of the LV tip obtained on postoperative day 1. The Hi setting was defined as 1 V greater than this anodal LV ring threshold at a fixed pulse-width. According to this convention, bipolar LV pacing at the Hi setting should result in capture of both the LV tip and LV ring and thus should afford the greatest chance of virtual electrode expansion. In keeping with experimental data, the anodal capture threshold of the LV ring was universally higher than the cathodal LV tip threshold. During follow-up periods, LV Capture Management was set on “monitor” to assure proper capture but to avoid reprogramming of LV output. Patients were excluded from further analysis or programming if any outputs determined by the above specifications resulted in phrenic nerve capture. Eligible patients were randomized to the Hi setting (all bipolar LV pacing) or the Lo setting (bipolar LV pacing or LV tip–to–right ventricular [RV] coil pacing) after implantation. All patients in the Hi group were programmed to LV bipolar pacing, thereby eliminating the potential for RV anodal capture. In those with LV tip–to–RV coil pacing in the Lo group, no cases of RV coil anodal capture were observed.

### Data Collection and Follow-Up

During implantation, intracardiac electrograms during Lo and Hi bipolar LV pacing were obtained, and RV tip–to–ring sensing was obtained to measure acute intracardiac conduction times. The interventricular conduction times (IVCTs) at both Lo and Hi settings were measured between the intracardiac LV stimulus marker and the onset of the bipolar RV electrogram. ECGs were obtained during Hi and Lo LV-only pacing in most cases to analyze surface ECG changes in paced QRS morphology between Hi and Lo settings. Postoperatively, pacing was programmed off for baseline measurements.

Baseline demographic data, 6-minute walk test results, Minnesota Living With Heart Failure (MLWHF) questionnaire responses, and transthoracic echocardiographic data were collected. Echocardiographic data included LV ejection fraction, LV end-diastolic volume, and LV end-systolic volume, as determined by Simpson's biplane method of discs. LV internal diameter in diastole and LV internal diameter in systole also were recorded. Finally, mitral regurgitation was quantified. Patients then were randomized by a third party not involved in data collection to the Hi-output arm or Lo-output arm. All patients underwent a single AV and VV optimization algorithm at enrollment with the use of previously published methods, which concentrated on maximizing the diastolic mitral inflow Doppler envelope for AV timing adjustments and maximizing aortic outflow envelopes for VV adjustments.^19^ Echocardiographers and researchers involved in data collection were blinded to LV output assignment. Patients were continued on medical therapy as determined by their treating physicians.

At 3 months, 6-minute walk distance, MLWHF score, and echocardiographic data were collected again. The LV stimulus intensity was reprogrammed to the alternate pacing arm output, and the second 3-month follow-up period ensued. At 6 months (3 months after the crossover), clinical data measurements were repeated and the study was concluded for that patient (Figure 1). At the conclusion of the study, LV output was reprogrammed according to the implanting physician's discretion.

### Statistical Analysis

The trial design resulted in paired data for each patient according to Hi or Lo LV settings. Statistical analysis was performed with SPSS (Version 16.0, SPSS Inc). All the study endpoints were continuous variables and were analyzed with paired or independent-samples *t* test as appropriate. The distribution of all study variables had a reasonably normal distribution on Q-Q plot and histogram analysis. New York Heart Association class variables, MLWHF scores at follow-up, and end-diastolic diameter at low-output setting did meet significance (*P*<0.05) on Shapiro-Wilk test of normality. For these variables, additional analysis with nonparametric Wilcoxon matched paired tests showed similar results. All data are expressed as mean±standard deviation. *P* values and confidence intervals were 2 tailed, and a *P* value <0.05 was considered significant.

## Results

A total of 60 patients were screened during CRT implantation between July 2010 and December 2010. Of these, 9 patients were excluded because of screen failure (5 patients had diaphragmatic stimulation, 3 patients had Hi setting measurements above the programmable output of the device, and in 1 patient the calculated Lo output was higher than the anodal ring threshold), and 1 patient withdrew. Fifty patients provided written informed consent and were enrolled in the study. Of these, 6 patients were lost to follow-up, 3 patients had diaphragmatic stimulation during follow-up and exited the study, 1 patient exited because of pregnancy, and 1 patient died secondary to worsening heart failure. A total of 39 patients successfully completed the study and were included in final data analysis.

The baseline demographic and clinical characteristics of the study group are provided in Table 1. All variables included in the endpoint analysis had a reasonably normal distribution. Of the 39 patients, 26 (66.7%) had left bundle-branch morphology, 6 (15.4%) had right bundle-branch morphology, and 7 (17.9%) were RV paced. The mean unipolar cathodal tip threshold was 1.1±0.7 V at 0.8±0.3 ms, and the mean unipolar anodal ring threshold was 4.3±1.6 V at 0.9±0.3 ms at implantation. Anodal ring threshold was found always to be higher than the cathodal tip threshold. Because of changes in the thresholds frequently noted on day 1 after implantation at the time of randomization, the Lo and Hi settings were ≍1 V above the cathodal and anodal thresholds measured intraoperatively. The average Lo setting was 2.4±0.9 V at 0.8±0.3 ms, and the average Hi setting was 5.8±1.7 V at 0.9±0.3 ms. RV-to-LV optimization at randomization resulted in the following programmed timing adjustments: 14 patients with RV output programmed ahead of LV, with a mean delay of 45.7±24.1 ms; 13 patients with LV output ahead, with a mean delay of 40±20 ms; and 12 patients with simultaneous activation.

**Table 1.** Baseline Characteristics

Age, y	66.4±9.8
Sex, male	71.8 (28/39)
Ischemic cardiomyopathy	53.8 (21/39)
Atrial fibrillation	30.8 (12/39)
Hypertension	79.5 (31/39)
Diabetes	35.9 (14/39)
QRS duration, ms	159.3±23.1
Ejection fraction, %	30.8±11.9
β-Blockers	100 (39/39)
ACE-I or ARB	79.5 (27/39)
Diuretics	69.2 (27/39)
Hydralazine	12.8 (5/39)
Long-acting nitrates	17.9 (7/39)
Statins	69.2 (27/39)
Amiodarone	5.1 (2/39)

Values are given as mean±standard deviation or percentage (n/N). ACE-I/ARB indicates angiotensin-converting enzyme inhibitor/angiotensin II receptor blocker; statins, 3-hydroxy-3-methyl-glutaryl-CoA reductase inhibitors.

Of the 39 patients who completed the study, 22 (56.4%) initially were randomized to the Lo arm. At follow-up, 2 patients (both in the Lo arm) had partial loss of capture according to LV capture management monitoring. The thresholds were 0.175 and 0.5 V higher than programmed settings, respectively. These 2 patients were included in the analysis on the basis of intention to treat.

**Figure 2.**
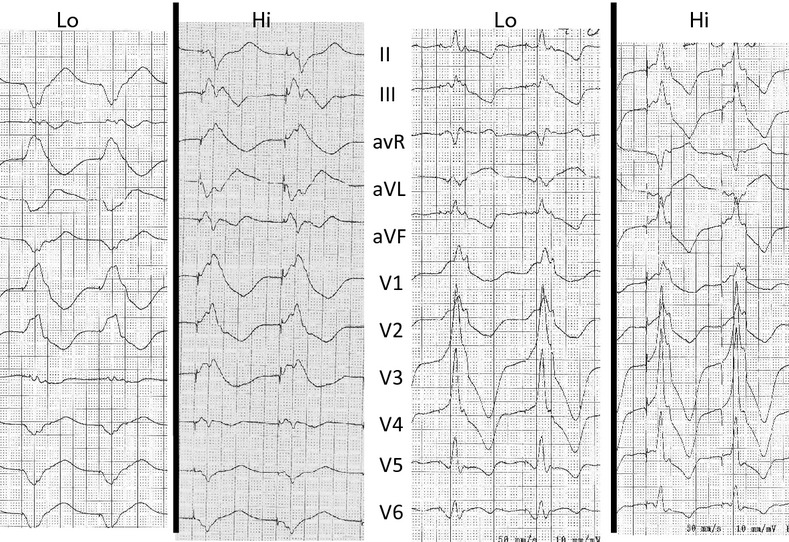
Differences on ECG between Lo and Hi LV-only bipolar pacing for 2 study participants. The QRS morphology differences are most notable in the frontal leads for the patient on left and in leads III, aVL, and aVF for the patient on right. (Lead I is not shown to protect subject identifiers).

Transventricular conduction times and 12-lead ECGs for both arms were analyzed. Although there were striking examples of shortened conduction times and visible changes on QRS morphology in the same study participant between Hi and Lo arms (Figure 2), as a group, no significant differences in IVCT were noted. Overall, 16 patients had a reduction in IVCT in the Hi arm as compared to the Lo arm. The mean improvement was 17.5±20.0 ms (*P*=NS).

Results for the clinical and echocardiographic endpoints between baseline and the 2 study arms are shown in Table 2. Both Hi and Lo arms had significant improvements in New York Heart Association class (Lo: 3±0 versus 2.4±0.5, *P*<0.01; Hi: 3.0±0 versus 2.4±0.6, *P*<0.001), 6-minute walk distance (Lo: 692±343 ft versus 995±368 ft, *P*<0.01; Hi: 699±346 ft versus 982±379 ft, *P*<0.001), and MLWHF (Lo: 43.3±24.0 versus 21.2±20.6, *P*<0.01; Hi: 43.3±24.0 versus 23.6±22.0, *P*<0.001) compared to baseline. End-diastolic diameter in the Lo arm and end-systolic diameter in the Hi arm also improved significantly as compared to baseline, and other echocardiographic parameters had nonsignificant trends toward improvement as compared to baseline in both trial arms.

**Table 2.** Results of Hi and Lo LV Pacing Arms Compared to Baseline and to Each Other

Parameters	Baseline vs Lo Output(n=39)	Baseline vs Hi Output(n=39 Unless Noted)	Lo Output vs Hi Output(n=39 Unless Noted)
Ejection fraction, %			
Mean (95% CI)	30.8 (26.9 to 34.6) vs 33.3 (29.9 to 36.7)	30.8 (26.9 to 34.6) vs 32.8 (28.8 to 36.8)	33.3 (29.9 to 36.7) vs 32.8 (28.8 to 36.8)
Difference (95% CI)	−2.5 (−6.9 to 1.9)	−2.0 (−6.1 to 2.1)	0.5 (−2.1 to 3.1)
*P*	0.249	0.325	0.704
End diastolic diameter, cm			
Mean (95% CI)	5.7 (5.5 to 6.0) vs 5.5 (5.1 to 5.8)	5.7 (5.5 to 6.0) vs 5.6 (5.3 to 5.9)	5.5 (5.1 to 5.8) vs 5.6 (5.3 to 5.9)
Difference (95% CI)	0.3 (0.1 to 0.5)	0.2 (−0.03 to 0.4)	−0.1 (−0.3 to 0.1)
*P*	<0.01	0.106	0.252
End systolic diameter, cm			
Mean (95% CI)	4.9 (4.6 to 5.3) vs 4.6 (4.3 to 5.0)	4.9 (4.6 to 5.3) vs 4.7 (4.3 to 5.0)	4.6 (4.3 to 5.0) vs 4.7 (4.3 to 5.0)
Difference (95% CI)	0.3 (0.1 to 0.6)	0.3 (0.1 to 0.6)	−0.1 (−0.3 to 0.1)
*P*	0.511	0.017	0.619
End diastolic volume, cm			
Mean (95% CI)	147.3 (129.6 to 165.1) vs 138.5 (120.2 to 156.9)	148.6 (130.5 to 166.6) vs 144.7 (126.3 to 163.1)	139.9 (121.3 to 158.6) vs 144.7 (126.3 to 163.1)
Difference (95% CI)	8.8 (−3.9 to 21.5)	3.9 (−10.2 to 17.9)	−4.8 (−16.7 to 7.2)
*P*	0.169	0.581, n=38*	0.426, n=38*
End systolic volume, mL			
Mean (95% CI)	105.6 (89.5 to 121.6) vs 96.4 (80.1 to 112.6)	107.3 (91.3 to 123.4) vs 100.6 (84.6 to 116.5)	98. 0 (81.7 to 114.3) vs 100.6 (84.6 to 116.5)
Difference (95% CI)	9.2 (−1.6 to 20.0)	6.8 (−5.1 to 18.7)	−2.6 (−10.5 to 5.3)
*P*	0.093	0.257, n=38*	0.514, n=38*
NYHA class			
Mean (95% CI)	3.0 (3.0 to 3.0) vs 2.4 (2.2 to 2.5)	3.0 (3.0 to 3.0) vs 2.4 (2.2 to 2.6)	2.4 (2.2 to 2.5) vs 2.4 (2.2 to 2.6)
Difference (95% CI)	0.7 (0.5 to 0.8)	0.6 (0.5 to 0.8)	0.0 (−0.1 to 0.0)
*P*	<0.01	<0.001	1.000
6-Minute walk distance, feet			
Mean (95% CI)	692.9 (581.5 to 804.4) vs 995.4 (876.1 to 1114.8)	699.5 (585.8 to 813.2) vs 982.0 (857.1 to 1106.9)	999.1 (876.7 to 1121.5) vs 982.0 (857.1 to 1106.9)
Difference (95% CI)	−302.5 (−387.9 to −217.1)	−282.5 (−365.7 to −199.4)	17.1 (−57.0 to 91.2)
*P*	<0.01	<0.001, n=38†	0.643, n=38†
MLWHF score			
Mean (95% CI)	43.3 (35.5 to 51.1) vs 21.3 (14.6 to 28.0)	43.3 (35.5 to 51.1) vs 23.6 (16.1 to 31.1)	23.6 (16.1 to 31.1) vs 22.1 (14.5 to 29.6)
Difference (95% CI)	22.1 (14.5 to 29.6)	19.7 (12.7 to 26.8)	−2.3 (−7.1 to 2.4)
*P*	<0.01	<0.001	0.331

CI indicates confidence interval; NYHA; New York Heart Association; and MLWHF, Minnesota Living With Heart Failure.

* This comparison excludes a single patient who had no recorded diameters at the end of Hi follow up.

† This comparison excludes a single patient who was unable to perform the 6-minute walk because of unrelated injury.

In contrast to the improvements from baseline, comparisons of variables between the Hi and Lo arms showed no significant differences or observable trends in echocardiographic results, MLWHF score, New York Heart Association class, and 6-minute walk distance. In a separate analysis, the same comparisons were performed on the subgroup of study participants in whom a decrease in IVCT was observed with Hi LV pacing. Despite the observed shortening of IVCT, no improvement in clinical or echo parameters was seen in this subgroup. A final analysis to account for the effects of time and order of randomization was performed. Clinical and echocardiographic parameters of patients enrolled to Lo settings in the first 3 months were compared to those enrolled to Lo settings in the second 3 months. The same comparison was repeated for the Hi setting. This analysis showed no significant (*P*>0.05) differences among groups, which supports that the sequence of randomization had no effect on the comparison of our experimental arms.

There were no significant differences in ejection fraction, end-systolic and end-diastolic diameters, or end-systolic and end-diastolic volumes in response to Hi and Lo pacing between ischemic (n=21) and nonischemic patients (n=18). There was also no significant different difference in the 6-minute walk distance.

The ischemic patients had significantly lower MLWHF scores than those of nonischemic patients with both Hi and Lo pacing (Lo group: 13.8±14.0 versus 29.9±24.0, *P*=0.013; Hi group: 15.3±14.9 versus 33.2±27.1, *P*=0.013). However, even at baseline, ischemic patients had significantly lower MLWHF scores (33.3±19.5 versus 54.9±24.1, *P*=0.004).

Finally, we considered the possibility that Hi pacing might affect only those who are “nonresponders” to CRT. Subset analysis of 10 patients who failed to respond echocardiographically to “Lo” LV pacing did not show significant improvement in any parameters when patients crossed over to the Hi arm.

## Discussion

Our data suggest that, despite preclinical evidence that increased LV stimulus intensity improves conduction times and myocardial contractility, there were no clinical advantages to increasing LV pacing output in patients who have standard indications for CRT. These findings are important for several reasons.

First, our results indicate that expansion of directly stimulated myocardium on the order of centimeters is insufficient to improve clinical response to CRT implantation. There has been much interest in altering LV lead location to the region of greatest delay to enhance CRT response.^9,20^ With our design, we were careful to choose a Hi setting that was sufficient for substantial expansion of the LV virtual electrode. The Hi setting produced simultaneous cathodal capture of the LV tip and anodal capture of the LV ring, which would alter the region of depolarization by a magnitude of ≍2 centimeters, based on the distance between the LV lead tip and ring electrodes.^21^ In almost all cases, this expansion occurred away from the apex of the LV, which has been shown to correlate with improved clinical response rates compared to more apical lead positions.^22^ Also, the change in depolarization between Hi and Lo settings was frequently apparent on a surface ECG during LV-only pacing, as seen in the example provided.

We considered that this change in the virtual electrode might affect only those with clear evidence of improvement in transventricular conduction with the Hi setting. Therefore, a subanalysis of patients who showed improvement in IVCT with Hi settings was performed. This likewise showed no clinical improvements in follow-up. These findings suggest that if altering LV virtual electrode size or position alters CRT response, the alteration would have to be relatively large and would be difficult to achieve within a single venous branch.

Second, our findings suggest that low programmed LV output to prolong battery life is feasible and that there is no additional clinical benefit of pacing at high output or of having a higher “safety margin” of LV lead capture. The monitored capture management algorithm also suggests that LV lead thresholds obtained on postoperative day 1 are relatively reliable and seem to be stable in the long term, inasmuch as only 2 of the 39 patients had capture loss detected at programmed settings of 1 V above LV tip threshold. These findings have valuable implications for the programming strategy of CRT devices and should aid in prolonging battery longevity.

### Limitations

This crossover trial used paired data comparisons to increase power to detect changes, but there is a chance that the trial had insufficient power to detect small improvements in the clinical parameters provided. Nevertheless, the fact that we observed significant improvements from baseline for both groups suggests that clinically relevant differences were detectable in our population. The protocol-derived and blinded LV ejection fractions of the study group at enrollment were occasionally higher than those determined in the clinical setting used to guide decision for CRT referral. This allowed some patients who had protocol-derived LV ejection fractions >35% to be enrolled in the study. Despite continuous capture-monitoring algorithms used in the study period, there still could have been transient loss of capture that was not recognized. Finally, this trial assessed increased LV stimulus intensity in the CRT-eligible population as a whole, and the effect this intervention has on the prespecified subgroup of nonresponders remains to be explored.

## Conclusions

The results of this prospective crossover trial indicate that low programmed LV output is clinically equivalent to higher outputs and should be used to prolong battery life in patients undergoing CRT implantation.
